# Genome sequencing and assessment of plant growth-promoting properties of a *Serratia marcescens* strain isolated from vermicompost

**DOI:** 10.1186/s12864-018-5130-y

**Published:** 2018-10-16

**Authors:** Filipe P Matteoli, Hemanoel Passarelli-Araujo, Régis Josué A Reis, Letícia O da Rocha, Emanuel M de Souza, L Aravind, Fabio L Olivares, Thiago M Venancio

**Affiliations:** 1 Laboratório de Química e Função de Proteínas e Peptídeos, Universidade Estadual do Norte Fluminense Darcy Ribeiro (UENF), Rio de Janeiro, Brazil; 2 Núcleo de Desenvolvimento de Insumos Biológicos para a Agricultura (NUDIBA), Universidade Estadual do Norte Fluminense Darcy Ribeiro (UENF), Rio de Janeiro, Brazil; 3 Departamento de Bioquímica e Biologia Molecular, Núcleo de Fixação Biológica de Nitrogênio, Universidade Federal do Paraná, Curitiba, Paraná Brazil; 4 National Center for Biotechnology Information, National Library of Medicine, National Institutes of Health, Bethesda, MD USA

**Keywords:** Comparative genomics, Soil microbiology, Whole-genome sequencing, Bioinformatics

## Abstract

**Background:**

Plant-bacteria associations have been extensively studied for their potential in increasing crop productivity in a sustainable manner. *Serratia marcescens* is a species of Enterobacteriaceae found in a wide range of environments, including soil.

**Results:**

Here we describe the genome sequencing and assessment of plant growth-promoting abilities of *S. marcescens* UENF-22GI, a strain isolated from mature cattle manure vermicompost. In vitro, *S. marcescens* UENF-22GI is able to solubilize P and Zn, to produce indole compounds (likely IAA), to colonize hyphae and counter the growth of two phytopathogenic fungi. Inoculation of maize with this strain remarkably increased seedling growth and biomass under greenhouse conditions. The *S. marcescens* UENF-22GI genome has 5 Mb, assembled in 17 scaffolds comprising 4662 genes (4528 are protein-coding). No plasmids were identified. *S. marcescens* UENF-22GI is phylogenetically placed within a clade comprised almost exclusively of non-clinical strains. We identified genes and operons that are likely responsible for the interesting plant-growth promoting features that were experimentally described. The *S. marcescens* UENF-22GI genome harbors a horizontally-transferred genomic island involved in antibiotic production, antibiotic resistance, and anti-phage defense via a novel ADP-ribosyltransferase-like protein and possible modification of DNA by a deazapurine base, which likely contributes to its competitiveness against other bacteria.

**Conclusions:**

Collectively, our results suggest that *S. marcescens* UENF-22GI is a strong candidate to be used in the enrichment of substrates for plant growth promotion or as part of bioinoculants for agriculture.

**Electronic supplementary material:**

The online version of this article (10.1186/s12864-018-5130-y) contains supplementary material, which is available to authorized users.

## Background

Composting and vermicomposting are widely known techniques that involve the stabilization of organic materials, with a concomitant sustainable production of valuable soil amendments [1]. Classical composting is defined as the biological decomposition of organic wastes, carried out by microorganisms in an aerobic environment [2], while vermicomposting also involves earthworms that promote aeration and help in waste stabilization by fragmenting the organic matter and boosting microbial activity [3]. Vermicomposting has a thermophilic stage, promoted by a thermophilic bacterial community that drives the most intensive decomposition step. This stage is followed by a mesophilic maturation phase that is largely mediated by earthworms and associated microbes [4]. Vermicomposted material holds greater amounts of total phosphorus (P), micronutrients and humic acid substances than the original organic material. In general, vermicomposts are considered a safe, cheap and rich source of beneficial microorganisms and nutrients for plants [5]. Further, bacteria isolated from vermicompost typically display greater saprophytic competence than those intimately associated with plants. From a biotechnological perspective, microbial survival and activity in the absence of a host plant represent an ecological advantage that can be used as a strategy to enrich substrates with nutrients, boosting plant growth and development (i.e. plant substrate biofortification) [6, 7].

Plants often benefit from mutualistic interactions with plant growth-promoting rhizobacteria (PGPR) [8]. PGPR can promote plant growth by various mechanisms, such as: 1) mitigation of abiotic stresses such as metal phytotoxicity [9], water or salinity stress [10]; 2) activation of defense mechanisms against phytopathogens [11]; 3) directly attacking pathogens [12]; 4) biological nitrogen fixation [13]; 5) solubilization of mineral nutrients (e.g. P and zinc, Zn) [14]; 6) production phytohormones [15] and; 7) secretion of specific enzymes (e.g., 1-aminocyclopropane-1-carboxylate deaminase) [16]. Due to their beneficial effects, there is a growing market for PGPR biofertilizers [17], which are based on bacteria of various genera, such as *Azospirillum*, *Bacillus*, and *Azotobacter* [18]. A notable example of successful application of PGPR in agriculture is the soybean (*Glycine max* L.) production in Brazil, in which the development and use of an optimized consortium of different strains of *Bradyrhizobium* sp. [19] led to very high productivity levels at significantly lower costs due to the virtually complete replacement of nitrogen fertilizers [20].

Knowledge of PGPR genomic content and plant interaction mechanisms has increased with the progress of second-generation sequencing technologies [21], which also allowed a number of comparative genomics studies. In a large-scale comparative analysis of alpha-, beta- and gamma-proteobacteria, Bruto et al. found no set of plant beneficial genes common to all PGPR, although the presence of certain genes could reflect the bacterial ecological type, such as the presence of *ppdC* (involved in auxin biosynthesis) exclusively in endophytic strains of *Azospirillum* and *Bradyrhizobium* [22]. *Bacillus amyloliquefaciens subsp. plantarum* FZB42 is a clear example of how genome mining strategies can uncover the genetic basis of plant growth-promoting capacity of a PGPR [23]. After promising results on auxin [24] and phytase [25] production in vitro, genome analysis also uncovered the molecular basis of how this strain exerts its antifungal [26], antibacterial [27] and nematicidal activities [28]. Another important example of genomic analysis of a PGPR is that of *Herbaspirillum seropedicae* SmR1, in which genes associated with nitrogen fixation and plant colonization were elegantly investigated [29].

*Serratia marcescens* is a Gram-negative and rod-shaped bacteria that has been proposed as a PGPR due to its P solubilization properties [30, 31], chitinase activity [32] and prodigiosin-mediated insect biocontrol [33]. *S. marcescens* has been described in association with several plants, such as cotton (*Gossypium hirsutum*) and maize (*Zea mays*) [34], rice (*Oryza sativa*) [35] and pinus (*Pinus pinaster*) [36]. *S. marcescens* FS14 (isolated from *Atractylodes macrocephala*) was shown to exert antagonistic effects against phytopathogenic fungi and genomic sequencing revealed the presence of an interesting pattern of secretion systems [37]. Some *S. marcescens* isolates have also been reported as opportunistic pathogens [38] and most comparative genomics studies of this species focused exclusively on its clinical relevance [39]. A comparative analysis of insect and clinical *S. marcescens* isolates revealed a substantial genetic diversity, as supported by a relatively low intra-species average nucleotide identity (ANI) of 95.1%. Further, a type II secretion system, often related to virulence [40], was found in the clinical but not in the insect strain [41].

Here we report a comprehensive characterization of *S. marcescens* UENF-22GI, a strain that has been shown to be abundant in mature cattle manure vermicompost, from where it was isolated. We performed a series of in vitro and in vivo experiments that show this bacterium’s ability to solubilize P and Zn, to synthesize indole compounds (likely the auxin indole acetic acid, IAA) and to counter the growth of phytopathogenic fungi. Inoculation with *S. marcescens* UENF-22GI increased maize growth and biomass under greenhouse conditions. Given its promising results as a PGPR, we sequenced its genome and carefully identified the genetic basis of these and other key features. The *S. marcescens* UENF-22GI genome also harbors an interesting horizontally-transferred genomic island involved in the production of a peptide antibiotic and in phage resistance via modification of DNA by a deazapurine base, which probably contributes to its competitiveness against other microorganisms. Further, phylogenetic reconstructions placed our strain in a clade almost exclusively comprised of non-clinical *S. marcescens* isolates. Collectively, our results strongly indicate that *S. marcescens* UENF-22GI is a good candidate to be used in inoculant formulations or as part of a strategy for biological enrichment of plant substrates.

## Results and discussion

### Identification of the isolate

During the initial characterization of abundant culturable bacteria from mature cattle vermicompost, we identified a notorious pigmented bacterium that was preliminarily characterized as *S. marcescens* by colony morphology, microscopy and 16S rRNA sequencing. This isolate was named *Serratia marcescens* UENF-22GI and was tested for a series of plant growth-promotion traits and, given the promising results, submitted it to whole-genome sequencing and comparative analysis.

### In vitro solubilization of P and Zn and synthesis of indole compounds by *S. marcescens* UENF-22GI

**Fig. 1:**
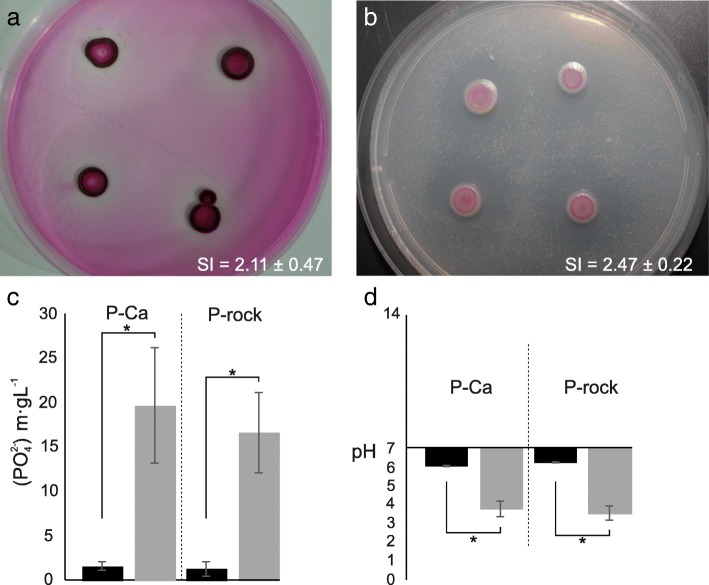
Phosphorus (**a**) and zinc (**b**) solubilization assays. Qualitative P and Zn solubilization assays were carried out with Ca_3_(PO_4_)_2_ (P-Ca) and ZnO as substrates, respectively. Halo formation around growing colonies was considered a positive result for solubilization. These results were used to compute the solubilization index (SI), which is the halo diameter divided by the colony diameter. Quantitative P solubilization assays were also performed using P-Ca or fluorapatite rock phosphate (P-rock) in the absence (black bars) or presence (gray bars) of *S. marcescens* UENF-22GI (**c**). pH variation in the culture media in the absence (black bars) or presence (gray bars) of *S. marcescens* UENF-22GI, indicating that P solubilization is probably driven by acidification (**d**). This assay was conducted in triplicates and statistical significance assessed by a by Student’s T-test (*P* < 0.05, indicated by asterisks)

We explored the capacity of *S. marcescens* UENF-22GI to solubilize P and Zn in vitro, as the availability of these elements is often a limiting factor in crop production [42]. We used the formation of a halo as a positive result for the solubilization of P and Zn, which are essential nutrients for bacterial growth. The halo and colony dimensions were also used to calculate a solubilization index (SI), which is useful to estimate the P and Zn solubilization capacities. Our results clearly show that *S. marcescens* UENF-22GI solubilizes P and Zn in vitro, with SI values of 2.47 ± 0.22 and 2.11 ± 0.47, respectively (Fig. 1a and b). We have also used a quantitative approach to measure P solubilization using two distinct inorganic P sources: calcium phosphate (P-Ca) and fluorapatite rock P (P-rock). Remarkably, we found that *S. marcescens* UENF-22GI increases the amount of soluble P by 12- and 13-fold with P-Ca and P-rock, respectively (Fig. 1c). Because acidification is a common P solubilization mechanism, we have also monitored pH variation and found striking media acidification patterns using glucose as carbon source, from 7.0 to 3.78 and 3.54 for P-Ca and P-rock, respectively (Fig. 1d). Although most P-solubilization screenings are conducted using only Ca-P, P is typically associated with Fe and Al in most tropical soils. The ability of *S. marcescens* UENF-22GI to solubilize P from P-rock is important, as this P source is recommended for organic agricultural systems. Hence, we propose that P-rock and *S. marcescens* UENF-22GI could be used in combination as a P-fertilization strategy for tropical soils.

**Fig. 2:**
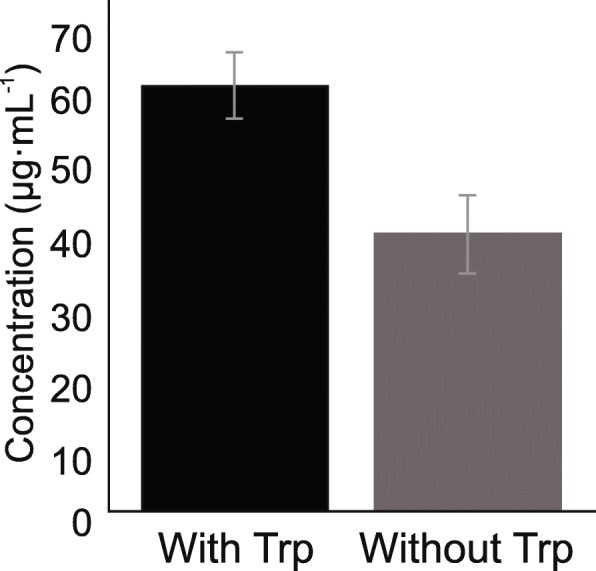
Biosynthesis of indole compounds in the presence and absence of tryptophan (Trp). An aliquot of the *S. marcescens* UENF-22GI inoculum was transferred to Dygs medium with or without tryptophan (100 mg∙L^− 1^) and incubated for 72 h in the dark, at 30 °C and 150 rpm. To evaluate indole synthesis, 150 μL of grown bacteria were transferred to microplates and 100 μL of Salkowski reagent (see methods for details) were added. The plate was incubated for 30 min in the dark and samples analyzed at 492 nm on a spectrophotometer

Bacterial production of phytohormones (e.g. indole-3-acetic acid, IAA, an auxin) is considered a major factor in enhancing plant growth [43]. IAA is a primary regulator of plant growth and development. Tryptophan-dependent IAA biosynthesis pathways have been reported in Bacteria, out of which the indole-3-acetamide (IAM), indole-3-acetonitrile (IAN), indole-3-pyruvic acid (IPyA) and tryptamine (TAM) pathways are well documented [44, 45]. Since the IAA biosynthesis genes are also involved in the Ehrlich pathway (degradation of amino acids via transamination, decarboxylation, and dehydrogenation), gene presence alone is not sufficient to determine IAA production. Therefore, we tested the ability of *S. marcescens* UENF-22GI to synthesize indole compounds in vitro and verified that it produces IAA either in the presence or in the absence of tryptophan, although greater IAA levels were observed in the former condition (Fig. 2).

### Biofilm formation and biocontrol of phytopathogenic fungi

**Fig. 3:**
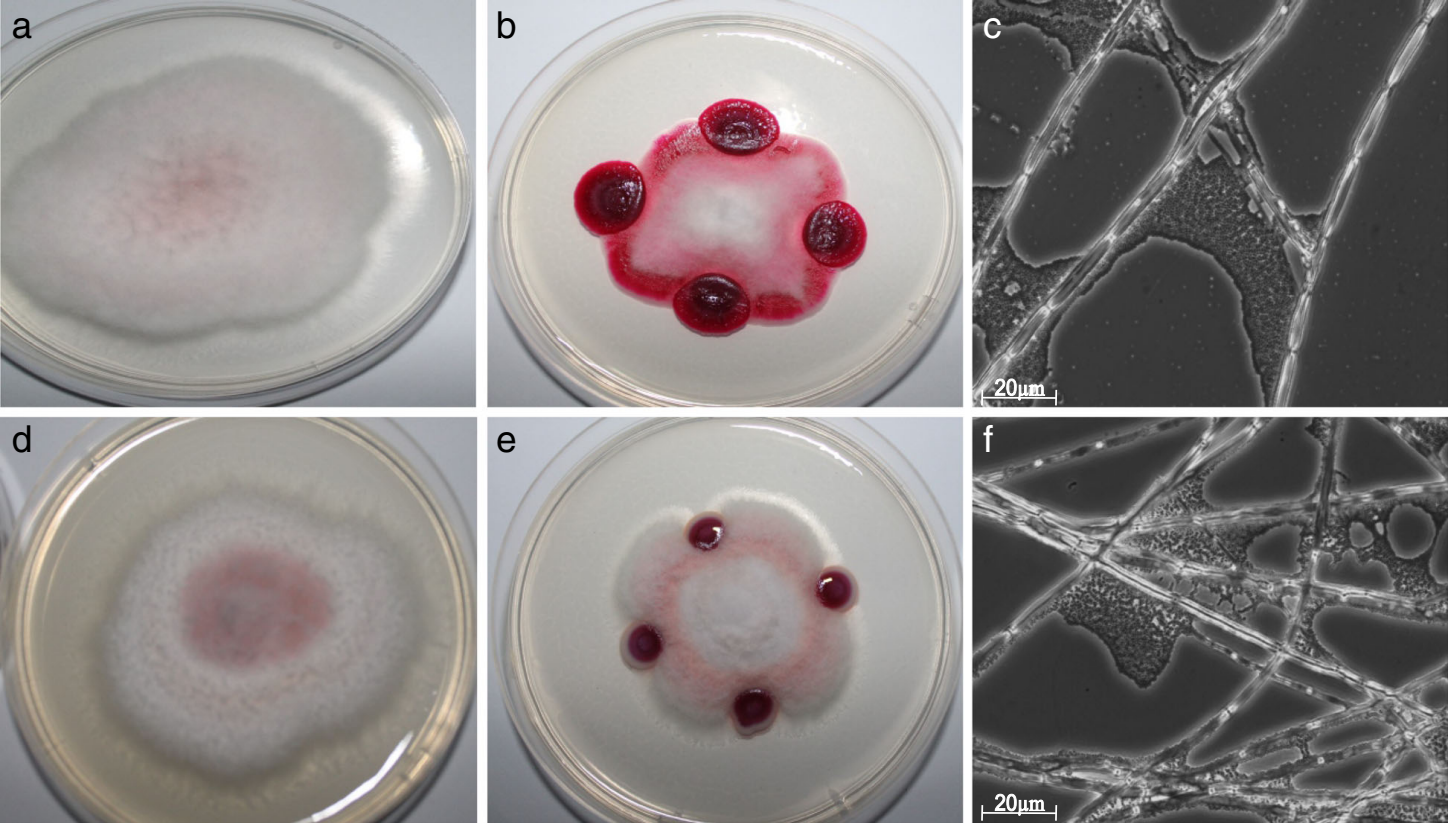
Dual growth assays of *S. marcescens* UENF-22GI and two phytopathogenic *Fusarium* species. Controls were conducted with *F. oxysporum* and *F. solani* grown without *S. marcescens* UENF-22GI (**a** and **d**, respectively). In the dual growth assays, *S. marcescens* UENF-22GI was placed in four points equidistant to the *F. oxysporum* and *F. solani* (**b** and **e**, respectively). The adherence of *S. marcescens* UENF-22GI to *F. oxysporum* and *F. solani* hyphae was demonstrated by optical microscopy (**c** and **f**, respectively)

Many fungi colonize the rhizosphere and must cope with strong competition from soil bacteria. Several of these fungi are phytopathogenic and pose serious risks to agriculture [46]. To assess the antifungal properties of *S. marcescens* UENF-22GI, we performed a dual growth assay and found that *S. marcescens* UENF-22GI counters the growth of *Fusarium oxysporum* and *F. solani* (Fig. 3). The strategy deployed by *S. marcescens* UENF-22GI to hinder fungal growth probably involves massive biofilm formation on *Fusarium* hyphae (Fig. 3; Additional file 1: Figure S1), which probably facilitates the colonization and degradation of fungal cell walls. In addition, there is a conspicuous delineation of the space occupied by *F. solani* by prodigiosin (Additional file 1: Figure S1), supporting the previously proposed antifungal activity of this secondary metabolite [47].

We have also performed a time-course dual growth experiment using *F. solani* and *S. marcescens* UENF-22GI for 12 days, which confirmed the results described above, indicating that *F. solani* does not outcompete *S. marcescens* UENF-22GI, even over a longer time period (Additional file 1: Figure S1). Importantly, *S. marcescens* UENF-22GI did not display any obvious negative effect on the growth of *Trichoderma* sp. (Additional file 1: Figure S1), a well-known plant growth-promoting fungus. Finally, the *S. marcescens* UENF-22GI ability to limit *F. solani* growth cannot be merely attributed to the physical occupation of the Petri dish, as a similar effect was not observed when *H. seropedicae*, a well-known PGPR, was used in the dual growth assays with *F. solani* (Additional file 1: Figure S1). Thus, we consider *S. marcescens* UENF-22GI a good candidate to be used in combination with *Trichoderma* sp. in inoculant formulations.

### *S. marcescens* UENF-22GI increases growth and biomass of maize seedlings

**Fig. 4:**
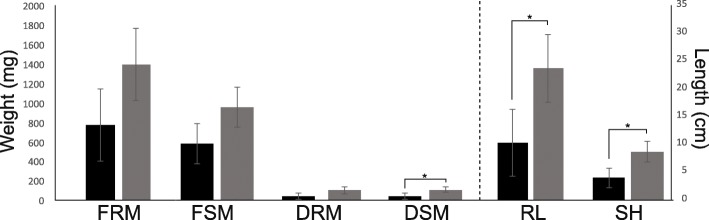
Effect of *S. marcescens* UENF-22GI inoculation on maize seedlings. Germinated seedlings (with 2 to 2.5 cm radicle root length) were transferred to glass tubes containing sterilized vermiculite (one seed per tube). Inoculation was performed by application of 1 mL of the *S. marcescens* UENF-22GI suspension (10^8^ cells∙mL^− 1^) over the seedlings (gray bars). Plants inoculated with 1 mL of the sterile Dygs medium were used as negative controls (black bars). The following metrics were recorded after 10 days: Fresh root mass (FRM), fresh shoot mass (FSM), dry root mass (DRM), dry shoot mass (DSM), root length (RL) and shoot height (SH). This assay was conducted in triplicates and statistical significance assessed by a by Tukey test (*P* < 0.05, statistical significance indicated by asterisks)

We conducted a pilot gnotobiotic experiment to evaluate whether *S. marcescens* UENF-22GI can promote plant growth, which is the overall effect of the beneficial properties of a PGPR on the host plant. The inoculation of plants can be performed using different methods (e.g. dipping, seed and soil inoculation) [18]. We evaluated the potential of *S. marcescens* UENF-22GI in enhancing maize growth in vivo by applying a suspension of *S. marcescens* UENF-22GI cells over maize seedlings and assessing their growth after 10 days (Fig. 4). Inoculation with *S. marcescens* UENF-22GI increased root and shoot mass (fresh and dry weight), as well as plant height and radicular length. The biomass increment was 100% in plant and root length, 80% for fresh root mass, 64% for fresh shoot mass and 150% for dry root and dry shoot mass when compared to the negative control (non-inoculated seedlings).

### Genome structure and comparative analysis

Given the interesting in vitro and in vivo results, we submitted the *S. marcescens* UENF-22GI genome to whole-genome sequencing using an Illumina HiSeq 2500 instrument (paired-end mode, 2 × 100 bp reads). Sequencing reads were processed with Trimmomatic and assembled with Velvet (see methods for details). The assembled genome consisted of 17 scaffolds (length ≥ 500 bp) encompassing 5,001,184 bp, with a 59.7% GC content and an *N*50 of 3,077,593 bp. The genome has 4528 protein-coding genes, 84 and 11 tRNA and rRNA genes, respectively (Additional file 1: Figure S2). We used BUSCO [48] to estimate genome completeness and detected the complete set of 781 *Enterobacteriales* single-copy genes, supporting the high quality and completeness of the assembled genome (Additional file 1: Figure S2). No plasmids were detected in the *S. marcescens* UENF-22GI genome by using plasmidSPADES and Plasmid Finder.

**Fig. 5:**
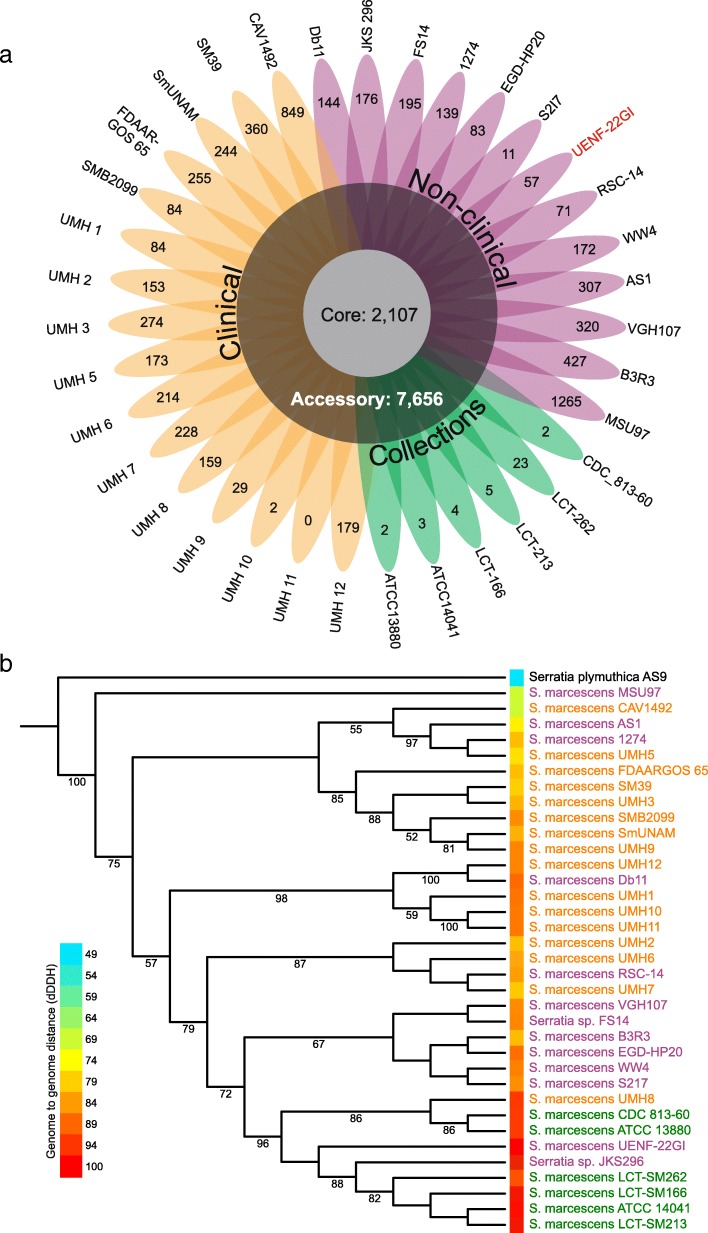
Comparative analysis of *S. marcescens* isolates. Clinical, non-clinical and collection isolates are represented in yellow, pink and green, respectively. **a** Flowerplot representing the pan-genome of 35 *S. marcescens* isolates. Labels on petal tips represent strain-specific genes. Numbers within colored petal areas represent strain-specific genes. **b** Multi-locus maximum likelihood tree reconstructed using concatenated alignment of ten single-copy core genes. Branch labels represent bootstrap support (in percentage; 1000 bootstrap replicates). The blue-to-red heatmap accounts for the distance of each isolate to *S. marcescens* UENF-22GI, estimated by the digital DNA:DNA hybridization (dDDH) method (d0 formula)

In order to understand genomic features at a species level, we computed the *S. marcescens* pan-genome. A pan-genome is defined as the entire gene repertoire of a given species [49]. We used 35 *S. marcescens* isolates with complete or scaffold-level genomes (Additional file 1: Table S1). A total of 16,456 gene families were identified, consisting of 2107 core genes shared by 100% of the isolates, 7656 accessory genes shared by more than one and less than 35 isolates and 57 genes unique to *S. marcescens* UENF-22GI (Fig. 5a; Additional file 1: Table S2). A recent study of 205 clinical strains from the United Kingdom and Ireland reported a pan-genome of 13,614 genes, 3372 core, and 10,215 accessory genes [39]. Interestingly, despite the greater number of strains in the clinical study, the reported pan-genome is smaller than that reported here, likely due to the greater diversity of the isolates considered in our study.

We further explored the pan-genome analysis to investigate the prevalence of antibiotic resistance genes, as part of a preliminary safety assessment of *S. marcescens* UENF-22GI. The pan-genome data were integrated with information from the CARD database (see methods for details). As expected, we found a number of antibiotic resistance genes in the *S. marcescens* core genome, including genes encoding a number of important efflux pumps (e.g. *MdtH*, *MsrB* and *AcrA-AcrB-TolC* multidrug efflux complex) [50]. Importantly, *S. marcescens* UENF-22GI lacks strain-specific antibiotic resistance genes, as well as a number of antibiotic resistance genes that are more prevalent in clinical than in non-clinical strains, such as, *emrE*, *mexG*, *mexQ*, *mexP*, *golS*, *qnrB31*, *qnrB37*, *aadA16*, *fosA2*, *fosA3*, *opmE*, *bla*_*OXA*-9_, *bla*_*TEM*-1_, and *sul1* [51, 52]. In particular, the latter seven genes are critical in clinical microbiology and have been associated with the emergence of resistant strains isolated from patients. We also noticed that several critical genes involved in the resistance against carbapenems, cephalosporins and monobactams were exclusively found in one clinical strain each (e.g. *bla*_KPC-1_, *bla*_SHV-30_, *bla*_IMP-1_ and *bla*_CMY-8_), thus belonging to the unique gene sets in the pan-genome analysis. This result is also supported by the absence of plasmids in *S. marcescens* UENF-22GI, as several of these critical genes (e.g. *bla*_KPC-1_ and *bla*_OXA-9_ and *aac(6′)-Ib*) are typically present in plasmids. Although follow-up experiments will be required to directly assess the safety of *S. marcescens* UENF-22GI to be applied in the field, our results show that this strain lacks key parts of the genetic signatures of multi-resistant clinical *S. marcescens* strains.

We also performed a phylogenetic reconstruction of the 35 strains included in the pan-genome analysis using ten single-copy core genes that were also present in the BUSCO reference set. This analysis revealed a clear separation of clinical and environmental isolates (Fig. 5b). The tree topology was also confirmed by whole-genome similarity metrics such as average nucleotide identity, average amino acid identity and genome-to-genome distance (Additional file 1: Table S3). A broader phylogenetic reconstruction using the 1815 core genes (out of 2107) that were also conserved in the clinical strains mentioned above [39] confirmed that the *S. marcescens* UENF-22GI clade is almost exclusively comprised of non-clinical strains (Additional file 1: Figure S3). Average nucleotide identity analysis also corroborated these results (Additional file 1: Figure S3). Strikingly, out of 219 clinical strains, only two (0.91%) belong to the *S. marcescens* UENF-22GI clade, suggesting that strains from this group rarely cause opportunistic (mostly nosocomial) infections, as observed with those from more distant clades. A similar approach has been recently used to better define the phylogenetic structure of the *Pantoea* genus [53].

Comparative genomic analysis helped us identify a genomic island that might contribute to the competitiveness of this species. We also carefully mined for genes potentially involved in the promotion of plant growth (Table 1) and integrated this list with the pan-genome data described above. These genes are grouped according to their general roles, namely: P and Zn solubilization, production of indole compounds (e.g. IAA) and spermidine, biofilm formation, pathogen competition and bioremediation. Interestingly, many genes are also present in several clinical strains (Additional file 1: Table S4), supporting previous observations that *S. marcescens* can thrive in different environments [38, 54]. Further, we provide strong quantitative support for previous studies reporting that prodigiosin production is a strong indicator of environmental strains [38]. Specifically, we found that the complete *pig* operon is present in only two clinical isolates out of 219 clinical isolates, exactly those belonging to the *S. marcescens* UENF-22GI clade (Additional file 1: Figure S3). The prevalence of the *pig* operon in non-clinical strains is probably related with the importance of prodigiosin in the competition with other microorganisms. We also identified other 91 genes that are conserved in all the strains from the *S. marcescens* UENF-22GI clade, but are absent in the other strains (Additional file 1: Table S5). This list comprises a number of transcriptional regulators and transporters that might be specifically related with the niches occupied by these strains. In the following sections we detail the characterization of the horizontally acquired Gap1 region and the annotation of genes potentially involved in plant-growth promotion.

**Table 1:** *Serratia marcescens* UENF-22GI genes associated with plant-growth promotion features discussed in this study

Annotation entry (AK961_)	Gene name
Phosphate and zinc solubilization	
07090, 07095, 07100, 07105, 07110	*pqqB*, *pqqC*, *pqqD*, *pqqE*, *pqqF*
10840	(PQQ)-dependent glucose dehydrogenase
17880	gluconolactonase
08395	2-gluconate dehydrogenase
17580, 17575, 17570	2-keto-gluconate dehydrogenase
21125, 21130, 21135, 21140, 21145	*phoU*, *pstB*, *pstA*, *pstC*, *pstS*
Tolerance against metal toxicity	
01055	arsenate reductase
03990	*arsR* family transcriptional regulator of *arsRBC* operon
03995	*arsB* arsenical pump membrane protein
04000	*arsC1* arsenate reductase
01905, 01915	copper resistance protein
09290	*copD*
07435, 07440	chromate transporter
12615	cobalt-zinc-cadmium efflux system
IAA and spermidine-related	
01575	*ipdC*
00655, 12310	auxin efflux carrier
18130,18125	*speA*, *speB*
18275, 18270	*speD*, *speE*
Biofilm formation	
20475, 20470, 20465, 20460,	*bcsA*, *bcsB*, *bcsC*, *bcsZ*
20480, 20485	*bcsQ*, *bcsR*
20490, 20495, 20500	*bcsE*, *bcsF*, *bcsG*
01650, 01655, 1660, 01665	*pgaA*, *pgaB*, *pgaC*, *pgaD*
13115	*adrA*
Biocontrol and resistance	
20530, 01270, 12935, 05475	*chiA*, *chiB*, *chiD*, *chiA1*
13300, 13305, 13310, 13315, 13320, 13325, 13330, 13335, 13340, 13345, 13350, 13355, 13360, 13365	*pigA*, *pigB*, *pigC*, *pigD*, *pigE*, *pigF*, *pigG*, *pigH*, *pigI*, *pigJ*,*pigK pigL*, *pigM*, *pigN*
16235	Kasugamycin resistance protein *ksgA*
03740, 14040	Bicyclomycin resistance protein
02590	Bicyclomycin multidrug efflux system
13395	Fosmidomycin resistance protein
07380	Barnase inhibitor
08005	Fusaric acid resistance protein

### Identification and analysis of the horizontally transferred Gap1 island

**Fig. 6:**
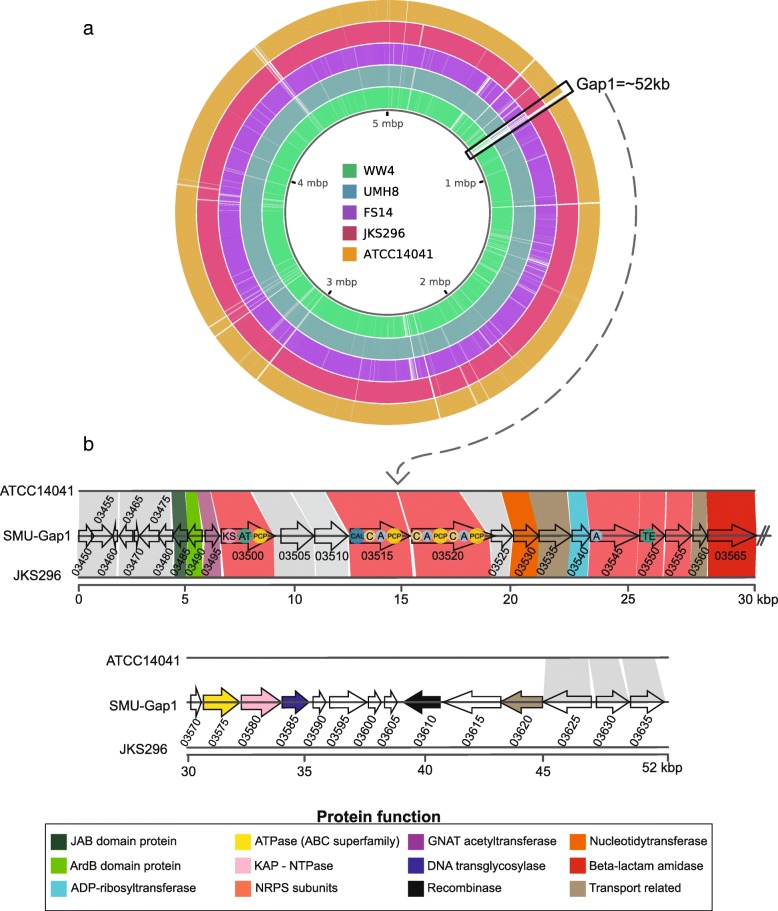
**a** Whole-genome alignment of *S. marcescens* UENF-22GI and some of the closest reference genomes. The black box indicates the horizontally-acquired region (Gap1); **b** Synteny analysis of part of the genes within the Gap1 region, emphasizing the presence of the NRPS-PKS domains: KS (ketosynthase), AT (acyltransferase), PCP (peptidyl carrier domain), CAL (coenzyme A ligase), C (condensation), A (adenylation) and TE (thioesterase). AK961_03495 encodes an antitoxin protein. AK961_03610 encodes an integrase that likely mediates the mobility of the element

Our comparative analysis uncovered a remarkable region in the *S. marcescens* UENF-22GI genome that is absent in most other *S. marcescens* genomes, which we named as Gap1 (Fig. 6a). Gap1 is partially conserved in the JSK296 and ATCC14041 strains (Fig. 6b), which belong to the *S. marcescens* UENF-22GI phylogenetic clade (Fig. 5b). Although partially eroded in several members of the clade, this result lends additional support to the greater proximity of *S. marcescens* UENF-22GI to a group of environmental strains (Fig. 6b). Case-by-case sequence analysis assisted by results from IslandViewer allowed us to predict that this ~ 52 Kb long genomic island contains 38 genes. This island encodes its own integrase of the tyrosine recombinase superfamily (AK961_03610), which is also encoded by several phages and bacterial mobile elements [55], suggesting that it supports its own genetic mobility. Genomic islands with closely related genes were also detected in several distantly related proteobacteria, such as *Erwinia piriflorinigrans* CFBP 5888*, Erwinia sp.* ErVv1*, Hahella sp.* CCB-MM4*, Enterobacter sp.* T1-1 and *[Polyangium] brachysporum*. The closest cognates of at least 15 genes (AK961_03495: AK961_03565) in this island are found in *Erwinia* species, raising the possibility of a relatively recent genetic exchange event involving *Serratia* and *Erwinia*.

We next investigated the island to identify genes potentially functioning as fitness determinants, which could explain this wide dissemination. At the 5′ flank of the island are two genes, respectively encoding a JAB domain protein of the RadC family and ArdB domain protein [56, 57] (AK961_03485, AK961_03490). These genes were recently identified as part of a system of proteins that enable mobile elements such as conjugative transposons and plasmids to evade restriction by host defense systems [56]. Comparable islands with genes coding for antibiotic biosynthesis and the anti-restriction genes have been previously described in the phytopathogenic *Erwinia* [58] and *Pantoea* [59] stains. The core of the island contains an operon, which is shared with the related islands that we detected in the above-stated bacteria, encoding the system predicted to synthesize a non-ribosomal peptide. The two largest genes (AK961_03515, AK961_03520) of this operon code for two giant multidomain non-ribosomal-peptide synthetases (NRPS), together with four predicted AMPylating domains that charge acyl groups and three condensation domains that ligate charged amino acids to form a peptide bond. Additionally, the operon contains a further gene encoding a standalone AMPylating enzyme and one for a thioesterase of the α/β-hydrolase fold (AK961_03545, AK961_03550). The last enzyme has been shown to be required for generation of a cyclic peptide in several NRPS systems [60]. Thus, the system encoded by Gap1 has the potential to synthesize a tetra- or penta-peptide skeleton with a possibly cyclic structure. Notably, the region also encodes a GNAT acetyltransferase (AK961_03495) that might either modify this peptide or confer auto-resistance against its toxicity. This operon also codes for a pol-β superfamily nucleotidytransferase (AK961_03530), which might modify the peptide generated by the NRPS by the addition of a nucleotide or regulate its production/secretion by nucleotidylation of one of the components of the system. The said operon codes for a predicted peptide transporter of the MFS superfamily (i.e. AK961_03535) that probably facilitates the export of the synthesized peptide out of the cell. Taken together, we interpret this NRPS system and associated proteins are generating an antimicrobial peptide.

We also found this island to encode a protein belonging to a previously unknown family of the ADP-ribosyltransferase (ART) fold (AK961_03540) [61]. Using sequence profile searches and profile-profile comparisons we showed that this novel family also includes the Pfam (“Domain of unknown function”) DUF4433 and the abortive phage infection protein AbiGi. Members of the ART superfamily either degrade NAD^+^ or transfer it to target substrates (e.g. proteins). Given the relationship to the AbiGi proteins, we predict that this protein might also play a role in anti-phage defense by means of its ADP-ribosyltransferase activity targeted either at self or viral proteins. In a similar vein, we also found an ATPase of the ABC superfamily (AK961_03575) that is related to AbiEii, another abortive infection protein involved in anti-phage systems [62]. More remarkably, the gene encoding this protein is also part of an operon coding for a KAP NTPase (AK961_03580) and another protein (AK961_03585), which are a version of an anti-phage system centered on these two proteins [63]. Of these the proteins, AK961_03585 is predicted to function as a novel DNA transglycosylase that is predicted to incorporate a modified base into DNA, likely a deazaguanine acquired from the queuine biosynthesis pathway [64].

Taken together, this island codes for multiple distinct fitness-promoting systems: one predicted to synthesize a potential antimicrobial peptide that could be deployed against competing organisms in compost. Further, it also encodes a beta-lactam amidase (AK961_03565) that could likely defend *S. marcescens* UENF-22GI against certain beta-lactams (e.g. penicillin) produced by competing bacteria. The further set of genes is likely to confer resistance against some bacteriophages and potentially enhance the fitness of this strain relative to other *Serratia* lacking the island. These fitness-conferring determinants carried by the Gap1 island and its cognates from other bacteria might have facilitated their dissemination by horizontal gene transfer.

### Phosphorus and zinc solubilization genes

As discussed above, in tropical environments, P is mostly present in poorly soluble mineral phosphates that are not readily available for plant uptake [65]. Microbial conversion of insoluble mineral P forms into soluble ionic phosphate (H_2_PO_4_^−^) is a key mechanism of increasing the P availability [66]. Further, the production and secretion of a variety of low molecular weight acids constitute a major strategy to solubilize not only P [65], but also Zn [67]. Among these substances, gluconic acid, produced by three oxidation reactions carried out by membrane-bound periplasmic proteins [68], is typically the most prominent.

The *S. marcescens* UENF-22GI genome harbors several genes involved in the production of gluconic acid from glucose (Table 1), which starts with the oxidation of glucose by a membrane-bound, periplasmic pyrroloquinoline-quinone (PQQ)-dependent glucose dehydrogenase (GDH; AK961_10840). The intermediate glucono-1,5-lactone is hydrolyzed to gluconate by a gluconolactonase (AK961_17880) and oxidized by 2-gluconate dehydrogenase (AK961_08395) to 2-ketogluconate, which is oxidized to 2-5-diketo gluconate by 2-keto-gluconate dehydrogenase, an enzymatic complex comprising a small (AK961_17580), a large (AK961_17575) and a cytochrome (AK961_17570) subunits (as in *Gluconobacter oxydans*, accession AB985494), encoded in the same operon. Gluconic acid synthesis requires the PQQ cofactor [69], which is produced by proteins encoded by the *pqqBCDEF* operon. Importantly, this operon is fully conserved in the *S. marcescens* UENF-22GI genome (genes AK961_07090: AK961_07110) (Table 1; Additional file 1: Figure S4). The *S. marcescens* UENF-22GI genome also has a conserved *pstABCS* operon (AK961_21130: AK961_21145) (Table 1; Additional file 1: Figure S4), which encodes a phosphate-specific transport system. Finally, current data indicate that Zn solubilization is largely carried out by the same genes involved in the solubilization of inorganic P [70]. Although the PQQ coenzyme is not exclusively related to P uptake, the co-occurrence of *pqq* with *gdh* and *pst* genes, along with the in vitro evidence described above, support that that *S. marcescens* UENF-22GI solubilizes P and Zn through soil acidification.

### Tolerance against metal toxicity

Successful soil bacteria often have to tolerate metal contamination, which can involve different strategies [71]. In addition, PGPR can alleviate the impact of heavy metals on plants by reduction, oxidation, methylation and conversion to less toxic forms [72]. We found a number of genes related to these roles in the *S. marcescens* UENF-22GI genome (Table 1): arsenate reductase (AK961_01055), *arsRBC* (AK961_03990, AK961_03995, AK961_04000), copper resistance protein (AK961_01905, AK961_01915, AK961_09290), *cusRS* (AK961_11430, AK961_11425), chromate transporter *chrA* (AK961_07435, AK961_07440), chromate reductase (AK961_21085) and *czcD* (AK961_12615). Although this list is likely incomplete due to the wide diversity of reactions and pathways involved in these tolerance pathways, our findings are in line with those from a recently sequenced genome of a *S. marcescens* strain that alleviates cadmium stress in plants [73]. Notably, a gene from the Gap1 island (AK961_03595) codes for a member of the YfeE-like transporter family, which transports chelated Fe/Mn and could potentially play a role in alleviating toxicity from these transition metals.

### IAA, spermidine biosynthesis and phenolic compound transport

We searched for IAA biosynthesis pathways in the *S. marcescens* UENF-22GI genome and found the *ipdC* gene (Table 1). This gene encodes a key enzyme responsible for the conversion of indole-3-pyruvate in indole-3-acetaldehyde, a critical step of the IPyA pathway. Disruption of *ipdC* dramatically decreases IAA production in *A. brasilense* [74]. In addition, we have also identified two putative auxin efflux carrier genes (AK961_00655, AK961_12310) (Table 1), suggesting that *S. marcescens* UENF-22GI also exports IAA. These results indicate that the IPyA pathway is active in *S. marcescens* UENF-22GI. Other IAA biosynthesis pathways were only partially identified and the genes pertaining to these pathways are also part of other processes. Hence, the activity of alternative pathways in *S. marcescens* UENF-22GI warrants further investigation.

In addition to IAA, we have also found the *speAB* (AK961_18130, AK961_18125) and *speDE* (AK961_18275, AK961_18270) operons, which are involved in spermidine biosynthesis (Table 1). Polyamines (e.g. spermidines) are essential for eukaryotic cell viability and have been correlated with lateral root development, pathogen resistance and alleviation of oxidative, osmotic and acidic stresses [75]. Therefore, spermidine production by *S. marcescens* UENF-22GI may constitute an additional mechanism involved in plant growth-promotion. Because spermidine may have other roles in bacterial physiology (e.g. biofilm formation) [76], future experiments are required to test if spermidine biosynthesis is directly involved in the *S. marcescens* UENF-22GI plant-growth promotion properties showed here.

Several plants produce phenolic compounds, which are part of their defense system and are also regulators of their own growth. Interestingly, the Gap1 island discussed above codes for a 4-hydroxybenzoate transporter (AK961_03620), which is closely related to cognate transporters from other plant-associated bacteria, such as *Pantoea ananatis*, *E. amylovora*, *Pseudomonas putida*, and *Dickeya* species. This suggests that this transporter might play a role in the plant-bacterium interaction via phenolic compounds such as benzoate, as has been proposed for certain *Xanthomonas* species [77]. Consistent with this, as in *Xanthomonas* species, we also found the key dioxygenases involved in utilization of the aromatic ring of phenolic compounds as well as the downstream enzymes of the oxoadipate pathway needed for further utilization of these compounds.

### Biofilm formation and biocontrol of phytopathogenic fungi

Bacterial biofilms are multicellular communities entrapped within an extracellular polymeric matrix [78] that are essential for survival, microbe-microbe interactions and root colonization [79]. We found several biofilm-related genes in the *S. marcescens* UENF-22GI genome (Table 1), such as *pgaABCD* (AK961_01650: AK961_01665) (Additional file 1: Figure S4). This operon is responsible for the production of poly-β-1,6-N-acetyl-D-glucosamine (PGA), which is associated with surface attachment, intercellular adhesion and biofilm formation in several species [80].

Cellulose is the fundamental component of plant cell walls and the most abundant biopolymer in nature. Cellulose biosynthesis has also been described in a broad range of bacteria and a variety of bacterial cellulose synthase operons are known [81]. In proteobacteria, cellulose biosynthesis is mainly carried out by the *bcsABZC* and *bcsEFG* operons, along with the *bcsQ* and *bcsR* genes, described as the *E. coli*-like *bcs* operon [82]. The *bcsABZC* (AK961_20475, AK961_20470, AK961_20465, AK961_20460) and *bcsEFG* (AK961_20490, AK961_20495, AK961_20500) operons are proximal to each other in the *S. marcescens* UENF-22GI genome, although in opposite strands (Additional file 1: Figure S4). The opposite orientation of these operons is also observed in others *S. marcescens* strains (e.g. WW4, B3R3 and UMH8), and might be related to the transcriptional regulation of biofilm synthesis in *S. marcescens*. Further, there are two regulatory genes upstream to the *bcsABZC* operon: *bcsQ* (AK961_20480) and *bcsR* (AK961_20485). These regulatory genes were also reported to be required for cellulose synthesis and subcellular localization of an active biosynthesis apparatus at the cell pole in γ-proteobacteria [83]. We have also found the *adrA* gene (AK961_13115), which encodes a diguanylate cyclase that synthesizes cyclic dimeric GMP, which binds to the BcsA and activates cellulose production [84]. BcsA has two cytoplasmic domains and transmembrane segments, while BcsB is located in the periplasm, anchored to the membrane; together they form the BcsAB complex, which functions as a channel for the addition of new residues to the nascent glucan molecule [81]. BcsC is an outer membrane pore [85] and BcsZ is an endoglucanase that may be involved in the alignment of β-glucans prior to export [86] or in the negative regulation of cellulose production [87]. The *bcsEFG* operon is also necessary for optimal cellulose synthesis [88] and its deletion disrupted cellulose production [89].

### Fungi biocontrol, prodigiosin production and resistance to antimicrobial compounds

Chitinases are central to the catabolism of chitin (i.e. poly β-(1- > 4)-N-acetyl-D-glucosamine), constituting a route by which bacteria can access a rich source of nutrients [90]. Chitinases break chitin into soluble oligosaccharides that can be transported into the periplasm via a chitoporin channel, where they are further processed into mono- and di-saccharides that are transported to the cytoplasm [91]. We found four chitinases in the *S. marcescens* UENF-22GI genome (Table 1); to further classify them we performed BLASTP searches on the Swissprot database. AK961_20530 shares 99% identity with chitinase A (accession: P07254), AK961_01270 shares 100% identity with chitinase B (accession: P11797), AK961_12935 shares 29% identity and 93% coverage with chitinase D (accession: P27050) and AK961_05475 shares 31% identity and 88% coverage with chitinase A1 (accession: P20533). We have also found other chitin metabolism genes in *S. marcescens* UENF-22GI, namely AK961_01260 and AK961_12890, which encode a chitin-binding protein and a chitobiase, respectively. Chitinases have received increased attention by the scientific community as a biocontrol mechanism deployed by several bacteria, including *S. marcescens* [32]. Bacterial chitinases can compromise fungal spore integrity and generate germ tube abnormalities [92]. Further, ChiA promotes the degradation of mycelia of several phytopathogenic fungi, including *Fusarium, Acremonium* and *Alternaria* species [93]. Chitinase applications are not restricted to fungal biocontrol and can also be deployed for bioremediation and bioconversion of chitin wastes, as well as part of an insect biocontrol strategies [94]. The presence these of chitinase together with prodigiosin production genes and our biocontrol results are in line with previous reports showing that *S. marcescens* chitinases may act synergistically with prodigiosin (and probably other molecules) to suppress fungal growth [54, 95].

As part of the arms race between microorganisms, several *Fusarium* species produce fusaric acid, a mycotoxin reported to be toxic to some microorganisms, such as *P. fluorescens* [96]. We observed that *S. marcescens* UENF-22GI has a gene (AK961_08005) that encodes a multi-TM protein of the FUSC solute exporter family, which has been demonstrated to provide resistance against fusaric acid in other bacteria. This might allow *S. marcescens* UENF-22GI to counter the fungal defenses and indirectly boost its fungicidal activity. We also detected the operon comprising genes involved in the biosynthesis of prodigiosin (i.e. the *pig* operon), the notorious red pigment, which is widely-conserved across several *Serratia* isolates [38]. Prodigiosin is most commonly found in environmental *S. marcescens* isolates and has been proposed to suppress growth of various fungi [47], bacteria [97], protozoans [98] and even viruses [99]. It has been recently suggested that prodigiosin has affinity for the lipid bilayer of the plasma membrane, causing outer membrane damage [100]. The *pig* operon in the *S. marcescens* UENF-22GI genome comprises 14 genes (Table 1; Additional file 1: Figure S4) arranged in a structure that resembles the *pig* operon from *S. marcescens* ATCC274 (also an environmental isolate) [101]. Our findings on the dual growth experiments indicate that that the *pig* operon is active and that prodigiosin delineates the growth area of *F. solani* (Fig. 3; Additional file 1: Figure S1).

*Streptomyces* species are ubiquitous in the soil and notable for the production of several antimicrobials [102]. Therefore, the presence of genes conferring resistance against these antimicrobials is a desirable feature of a successful PGPR. *S. marcescens* UENF-22GI produces several resistance proteins against *Streptomyces* antimicrobials such as bicyclomycin (AK961_03740, AK961_14040, AK961_02590), fosmidomycin (AK961_13395) and kasugamycin (AK961_16235). In addition, it also exhibits a type VI secretion system (T6SS) (AK961_04125-AK961_04210) that can mediate interbacterial antagonistic interactions [103, 104].

## Conclusions

In the present work, we described a thorough investigation of a *S. marcescens* UENF-22GI, abundant in mature cattle vermicompost. Previous studies have shown beneficial effects of other *S. marcescens* isolates on plants, such as in the mitigation of salt stress in wheat [105], in soil phytoremediation [106] and in ginger growth promotion [107]. We assessed the plant growth-promoting properties of *S. marcescens* UENF-22GI using in vitro biochemical assays and in vivo experiments in greenhouse conditions. Specifically, we found that this bacterium is able to: 1) solubilize inorganic P and Zn; 2) produce indole compounds; 3) counter the growth of two phytopathogenic *Fusarium* species by a combination of physical (i.e. biofilm formation) and biochemical (e.g. prodigiosin, chitinase) properties and; 4) increase growth and biomass of maize seedlings.

Given these interesting properties, we sequenced the *S. marcescens* UENF-22GI genome and carefully mined the genes that are likely responsible for these traits. Interestingly, the genome also harbors a mobile genomic island comprising 38 genes that were horizontally transferred. This region codes for a NRPS system and other proteins predicted to confer fitness advantage by various mechanisms, including DNA-modification and anti-phage defenses. Phylogenetic analyses using either a multi-locus approach with ten genes or 2107 core genes show that *S. marcescens* UENF-22GI groups with high statistical support within a clade comprised almost exclusively of non-clinical isolates. Together with the absence of critical antibiotic resistance genes that are more prevalent in clinical strains, the lack of plasmids and the presence of the complete prodigiosin biosynthesis operon, present in only two out of 219 clinical strains, we hypothesize that *S. marcescens* UENF-22GI is non-pathogenic to humans. A similar partial separation of pathogenic and non-pathogenic strains was also observed in *Burkholderia* and *Paraburkholderia*, respectively [108]. In *Pantoea agglomerans* strains, it has proven hard to define phylogenetic clades separating biocontrol strains from those reported as clinical isolates, although several lines of evidence support the misidentification of most (if not all) of the arguably clinical *P. agglomerans* isolates [109, 110]. Therefore, phylogenetic analysis like those reported here and elsewhere can help assess the potential of plant growth-promoting bacteria, in particular because many genera comprising well-known PGPR also have opportunistic pathogenic strains. In the future, it will be critical to conduct a more comprehensive phylogenetic analysis and comparative genomic study of *Serratia*, integrating additional data from in vitro and in vivo screenings, like those reported over the past few years for *P. agglomerans* [111], *Burkholderia* [112] and *Pseudomonas* [113]. Nevertheless, basic safety issues must be directly addressed before biotechnological applications of *S. marcescens* UENF-22GI can be envisaged. Collectively, our results add important information regarding *S. marcescens* plant growth-promoting abilities that can inspire future applications in inoculant formulations.

## Methods

### Vermicompost maturation

Mature vermicompost was produced with dry cattle manure as substrate inside a 150 L cement ring. Humidity was kept at 60-70%, by weekly watering and mixing. After 1 month, earthworms (*Eisenia foetida*) were introduced at the rate of 5 kg∙m^3^. After 4 months, earthworms were removed and the vermicompost was placed in plastic bags and stored at 25°. At the final maturation stage, the chemical composition of the substrate (in g∙kg^− 1^) was as follows: total nitrogen (1.9 ± 0.4); total carbon (22.99 ± 3.3); P_2_O_5_ (6.97 ± 1.4); C/N ratio of 13.8 ± 0.4 and pH (H_2_O) = 6.6 ± 0.18.

### Bacterial isolation and DNA purification

Serial dilutions were performed on a solution prepared by adding 10 g of vermicompost in 90 mL of saline (8.5 g∙L^− 1^ NaCl), followed by shaking for 60 min. Next, 1 mL of the initial dilution (10^− 1^) was added to a new tube containing 9 mL of saline (10^− 2^), and successively until 10^− 7^ dilution. Then, 100 μL of the final dilutions from 10^− 5^ to 10^− 7^ were taken and spread on plates containing solid Nutrient Broth (NB) with 8 g∙L^− 1^ of NB and 15 g∙L^− 1^ of agar in 1 L of distilled water. After incubation at 30 °C for 7 days, different colony types could be identified and, for purification, individual colonies were transferred to Petri plates with Dygs solid media [114] containing 2 g∙L^− 1^ of glucose, 2 g∙L^- 1^ of malic acid, 1.5 g∙L^− 1^ of bacteriological peptone, 2 g∙L^− 1^ of yeast extract, 0.5 g∙L^− 1^ of K_2_HPO_4_, 0.5 g∙L^− 1^ of MgSO_4_∙7H_2_O, 1.5 g∙L^− 1^ of glutamic acid and 15 g∙L^− 1^ of agar, adjusted to pH 6.0; these supplies were acquired from Vetec (São Paulo, Brazil). From the last dilution (10^− 7^) and after the isolation and purification on Dygs solid medium, a pink-to-red, circular, pulvinate elevation, punctiform and smooth surface bacterial colony was selected. Phase contrast microscopy revealed the presence of Gram-negative, rod-shaped and non-motile cells. This distinctive isolate, named UENF-22GI, was stored in 16 mL glass flask containing 5 mL of Nutrient Broth solid medium covered with mineral oil and later grown in liquid Dygs medium under rotatory shaker at 150 rpm and 30 °C for 36 h to perform in vitro and in vivo assays. Total DNA of UENF-22GI was extracted using QIAamp® DNA Mini Kit (QIAGEN GmbH, Hilden, Germany). DNA quantification and quality assessment were performed using an Agilent Bioanalyzer 2100 instrument (Agilent, California, USA).

### Phosphorus and zinc solubilization

Bacterial inocula were grown for 36 h on liquid Dygs media at 150 rpm and 30 °C until approximately 10^8^ cells.mL^− 1^ (O.D._540nm_ = 1.0) [114]. To carry out a qualitative P solubilization assay, 10 μl of the bacterial suspension were added to petri dishes containing 10 g∙L^− 1^ of glucose, 5 g∙L^− 1^ of ammonium chloride (NH_4_Cl), 1 g∙L^− 1^ of sodium chloride, 1 g∙L^− 1^ of magnesium sulfate heptahydrate (MgSO_4_∙7H_2_O), 15 g∙L^− 1^ of agar in 1 L of distilled water at pH 7.0, and incubated at 30 °C for 7 days. Two mineral P sources were tested: calcium phosphate Ca_3_(PO_4_)_2_ (P-Ca) and fluorapatite rock phosphate Ca_10_(PO_4_)_6_F_2_ (P-rock), both at 1 g∙L^− 1^. Positive P solubilization phenotypes were based on halo formation around bacterial colonies and results were expressed in the form of a Solubilization Index (SI), calculated as the halo diameter (d1) divided by the colony diameter (d2). The SI values can be used to classify the solubilization ability of a strain as low (SI < 2), intermediate (2 < SI < 4) and high (SI > 4) [115].

Quantitative P solubilization assays were also performed. 50 μL bacterial suspensions in Dygs liquid medium were transferred to 30 mL test tubes containing Pikovskaya liquid medium at pH 7.0, supplemented with P-Ca or P-rock at 1 g∙L^− 1^. The assay was carried out in orbital shaker at 150 rpm at 30 °C. After 7 days, a 5 mL aliquot was harvested and centrifuged at 3200 rpm for 15 min. The supernatant was used to determine the pH and to quantify soluble P levels by the colorimetric ammonium molybdate method (λ = 600 nm). Results were expressed in mg of PO_4_^2−^∙L^− 1^.

Zn solubilization was evaluated using 10 μL aliquots taken from the bacterial suspension and dropped onto petri dishes containing solid media [116] constituted of 10 g∙L^− 1^ of glucose, 1 g∙L^− 1^ of ammonium sulfate ((NH_4_)_2_SO_4_), 0.2 g∙L^− 1^ of potassium chloride (KCl), 0.1 g∙L^− 1^ of dipotassium phosphate (K_2_HPO_4_), 0.2 g∙L^− 1^ of magnesium sulfate heptahydrate, 1.0 g∙L^− 1^ of zinc oxide (ZnO), 15 g∙L^− 1^ agar, 1 L distilled water; the medium was incubated for 7 days at 30 °C. Zn solubilization was also assessed by halo formation around bacterial colonies. Both, Zn and P solubilization assays were carried out in triplicates.

### Production of indole compounds

To quantify the production of indole compounds, previously grown bacteria were transferred to glass tubes containing 5 mL of Dygs medium with or without tryptophan addition (100 mg∙L^− 1^), followed by 72 h incubation in the dark, at 30 °C and 150 rpm. To evaluate indole synthesis [117], 150 μL of grown bacteria were transferred to microplates and 100 μL of Salkowski reagent, which was prepared by diluting 1 mL of an iron trichloride hexahydrated (FeCl_3_∙6H_2_0) aqueous solution at 92.5 g∙L^− 1^ in 50 mL of perchloric acid (HClO_4_) 350 g∙L^− 1^ in water. The plate was incubated for 30 min in the dark and samples analyzed at 492 nm on a UV mini 1240 spectrophotometer (Shimadzu, Japan). This assay was conducted in triplicate.

### In vitro dual culture assays

In vitro bacterial-fungal dual culture assays were performed in 9 cm diameter Petri dishes containing Potato Dextrose Agar solid medium. A 5 mm diameter disk taken from the edge of actively growing hyphae of *F. solani* and *F. oxysporum* were inoculated at the center of each Petri dish. Suspensions of *S. marcescens* UENF-22GI were spotted in four equidistant quadrant points to the inoculated fungal disk. Control treatments (fungus only) were conducted in parallel to monitor fungal growth. Treatments were carried out for 10 days and three independent replicates were performed. In addition, time-course dual culture experiments were also performed for 12 days with *S. marcescens* UENF-22GI and *F. solani* or *Trichoderma* sp., a plant growth-promoting fungus. We have also tested *F. solani* in dual growth assays with *H. seropedicae* HRC54, a well-known PGPR without known anti-fungal properties. Samples from the transition zones between fungi structures and spotted bacteria were mounted on a glass slide and coverslip, observed under phase-contrast inverted optical microscope Zeiss Axio 10 Observer A1 and photodocumented with an Axiocam MRC 5 digital camera. For the time-course assays between *F. solani* and *S. marcescens* UENF-22GI (1 - 12 days of growth), bacterial inocula were spotted in three equidistant quadrant points to the inoculated fungal disk.

### In vivo plant-growth promotion assays

Maize (*Zea mays* var. UENF/506-11) seeds were surface-disinfected using ethanol 70% for 30 s, followed by a wash with 5% sodium hypochlorite (NaClO) for 20 min. Next, seeds were washed five times with sterile distilled water under stirring for 3 min and transferred to petri dishes containing 1.5% solidified agar for pre-germination for 4 days. Seedlings with 2.0 to 2.5 cm radicle length were carefully transferred under flow chamber to glass tubes of 2 cm diameter and 20 cm height containing 10 g of sterilized vermiculite (one seedling per tube). Meanwhile, the bacterial inoculum was prepared by growth in Dygs liquid media for 36 h, at 30 °C and 120 rpm. Inoculation was performed by application of 1 mL of the *S. marcescens* UENF-22GI suspension (10^8^ cells∙mL^− 1^) over the seedlings. Plants inoculated with 1 mL of sterile Dygs medium were used as negative controls. The assay was carried out under laboratory conditions with the average temperature at 30 °C and 12 h of light/dark photoperiod. After 10 days, plants were collected and the following biometric measurements were registered: shoot height (cm), total radicular length (cm), fresh root mass (mg), fresh shoot mass (mg), dry root mass (mg) and dry shoot mass (mg). This assay was performed in four replicates. Statistical analyses were performed using the SAEG software (Universidade Federal de Viçosa, Brazil) and obtained means were compared with the Tukey test.

### Genome sequencing and assembly

Paired-end libraries were prepared with the TruSeq Nano DNA LT Library Prep (Illumina) and sequenced on a HiSeq 2500 instrument at the Life Sciences Core Facility (LaCTAD; UNICAMP, Campinas, Brazil). The quality of the sequencing reads (2 × 100 bp) was checked with FastQC 0.11.5 (https://www.bioinformatics.babraham.ac.uk/projects/fastqc/). Quality filtering was performed with Trimmomatic 0.35 [118] and only reads with average quality greater than 30 were used. The *S. marcescens* UENF-22GI genome was assembled with Velvet 1.2.10 [119], with the aid of VelvetOptimiser 2.2.6 [120]. Scaffolding was performed with SSPACE 3.0 with default parameters [121]. QUAST 4.0 [122] was used to assess general assembly statistics. Genome completeness was assessed with BUSCO 3.0 [48], using the *Enterobacteriales* dataset as reference.

### Genome annotation and phylogenetic analysis

The assembled genome was annotated with the NCBI Prokaryotic Genome Annotation Pipeline [123]. Some annotations were manually improved with primary literature information and specific searches using BLAST [124] and Kegg Orthology And Links Annotation (BlastKOALA) [125]. The presence of plasmids was assessed with plasmidSPAdes 3.10 [126] and PlasmidFinder 1.3 [127]. Genes and operons involved in antibiotic and secondary metabolism were predicted using antiSMASH 4.0 [128]. Genes involved in antibiotic resistance were predicted by BLAST searches against the CARD database 2.01 [129], using 50% and 80% identity and coverage thresholds, respectively. The *S. marcescens* UENF-22GI genome was deposited on Genbank under the BioProject PRJNA290503.

Whole genome comparisons were conducted using BRIG 0.95 [130] and synteny was assessed using Synima v 1.0 [131]. Horizontal gene transfer regions were inferred with IslandViewer4 [132], followed by manual adjustments. Pan-genome analysis was performed with BPGA 1.3.0 [133]. Phylogenetic reconstructions were carried out using the predicted proteins of ten core housekeeping genes that were also present in the BUSCO’s reference dataset. Protein sequences were aligned using MUSCLE 3.8.31 [134] and evolutionary model selected with protest 3.4.2 [135]. Maximum-likelihood phylogenetic reconstructions were performed using RAxML 8.2.10 [136], with the Le and Gascuel model [137], gamma correction, SH local support and 1000 bootstrap replicates. Phylogenetic reconstructions using the core genome was performed using the same approach. Genomic distance patterns were computed with the digital DNA:DNA hybridization (dDDH) method, using whole genome formulae, average nucleotide identity and average amino acid identity [138]. The resulting phylogenetic tree and dDDH values were integrated and rendered in iTOL 3 [139].

## Additional file

Additional file 1: **Figure S1.** Time-course (24, 48, 120 and 288 h) dual growth of *S. marcescens* UENF-22GI with the phytopathogenic *Fusarium solani* on potato dextrose agar (PDA) solid medium. Note that *F. solani* colony growth (i.e. spread) was reduced by *S. marcescens* UENF-22GI, which in contrast was not significantly affected by the fungus, despite some alterations in the pigmentation patterns. At the bottom line, we show that *S. marcescens* UENF-22GI does not counter the growth of the beneficial saprophytic fungus *Trichoderma* sp.. We also observed a depigmentation of the *S. marcescens* UENF-22GI colony and its spread on the plate surrounding *Trichoderma* sp.. Finally, we used another bacteria species, *Herbaspirillum seropedicae*, to demonstrate that the *S. marcescens* UENF-22GI effects on *Fusarium* are not spurious or merely due to physical occupation of the Petri dish. **Figure S2.** General genomic features of *S. marcescens* UENF-22GI. **a)** Total length, number of protein-coding, tRNA and rRNA genes are represented, as well as the GC skew across the genome; **b)** BUSCO genome completeness assessment using 781 single-copy genes from the *Enterobacteriales* reference dataset. **Figure S3. a)** Maximum likelihood phylogenetic tree reconstructed with the alignments of the protein products of the 1815 core genes identified using 238 *S. marcescens* isolates. The tree was built with FastTree 2.1 (https://doi.org/10.1371/journal.pone.0009490). Branch labels represent SH local support values. The purple shaded box delimits the clade containing *S. marcescens* UENF-22GI and is mostly comprised of non-clinical strains; **b)** Clustering analysis of *S. marcescens* strains using Average nucleotide identity (ANI). This analysis also supports that *S. marcescens* UENF-22GI belongs to a mostly non-clinical clade. **Figure S4.** Plant growth-promoting operons found in the *S. marcescens* UENF-22GI genome. **a)** biosynthesis of pqq; **b)** phosphate transport system; **c)** poly-beta-1,6-N-acetyl-glucosamine biosynthesis; **d)** bacterial cellulose biosynthesis; **e)** prodigiosin biosynthesis; **f)** type VI secretion system (genes of unknown functions are in gray). **Table S1.** List of genomes used in this study. **Table S2.** List of unique genes in the *S. marcescens* UENF-22GI genome. **Table S3.** Whole-genome similarity metrics of *S. marcescens* genomes used in this study. The table contains the following metrics: average nucleotide identity, average amino acid identity and digital DNA:DNA hybridization (dDDH). **Table S4.** Presence/absence profiles of potential plant-growth promotion genes across *S. marcescens* strains. **Table S5.** List of genes conserved in all the strains from the *S. marcescens* UENF-22GI and absent in the other strains used in the pan-genome analysis. (ZIP 13900 kb)

## Abbreviations

ANI: Average nucleotide identity
dDDH: digital DNA:DNA hybridization
IAA: Indole-3-acetic acid
IPyA: Indole-3-pyruvic acid
NRPS: Non-ribosomal-peptide synthetases
PGPR: Plant growth-promoting rhizobacteria
PQQ: Pyrroloquinoline-quinone
SI: Solubilization index
Trp: Tryptophan
