# Supplementary material for: Genome sequencing and assessment of plant growth-promoting properties of a Serratia marcescens strain isolated from vermicompost
Source: BMC Genomics. 2018 Oct 16;19:750. doi: 10.1186/s12864-018-5130-y (PMC6192313; doi:10.1186/s12864-018-5130-y)
Supplement: Supplementary file 1 — Figure S1. Time-course (24, 48, 120 and 288 h) dual growth of S. marcescens UENF-22GI with the phytopathogenic Fusarium solani on potato dextrose agar (PDA) solid medium. Note that F. solani colony growth (i.e. spread) was reduced by S. marcescens UENF-22GI, which in contrast was not significantly affected by the fungus, despite some alterations in the pigmentation patterns. At the bottom line, we show that S. marcescens UENF-22GI does not counter the growth of the beneficial saprophytic fungus Trichoderma sp.. We also observed a depigmentation of the S. marcescens UENF-22GI colony and its spread on the plate surrounding Trichoderma sp.. Finally, we used another bacteria species, Herbaspirillum seropedicae, to demonstrate that the S. marcescens UENF-22GI effects on Fusarium are not spurious or merely due to physical occupation of the Petri dish. Figure S2. General genomic features of S. marcescens UENF-22GI. a) Total length, number of protein-coding, tRNA and rRNA genes are represented, as well as the GC skew across the genome; b) BUSCO genome completeness assessment using 781 single-copy genes from the Enterobacteriales reference dataset. Figure S3. a) Maximum likelihood phylogenetic tree reconstructed with the alignments of the protein products of the 1815 core genes identified using 238 S. marcescens isolates. The tree was built with FastTree 2.1 (https://doi.org/10.1371/journal.pone.0009490). Branch labels represent SH local support values. The purple shaded box delimits the clade containing S. marcescens UENF-22GI and is mostly comprised of non-clinical strains; b) Clustering analysis of S. marcescens strains using Average nucleotide identity (ANI). This analysis also supports that S. marcescens UENF-22GI belongs to a mostly non-clinical clade. Figure S4. Plant growth-promoting operons found in the S. marcescens UENF-22GI genome. a) biosynthesis of pqq; b) phosphate transport system; c) poly-beta-1,6-N-acetyl-glucosamine biosynthesis; d) bacterial cellulose bio [file 12864_2018_5130_MOESM1_ESM.zip › additionalFile1.pdf]

ADDITIONAL FILE 1

Genome sequencing and assessment of plant growth-promoting properties of a *Serratia marcescens* strain isolated from vermicompost

Filipe P. Matteoli[a], Hemanoel Passarelli-Araujo[a], Régis Josué A. Reis[b], Letícia O. da Rocha[b], Emanuel M. de Souza[c], L. Aravind[d], Fabio L. Olivares[b#] and Thiago M. Venancio[1a#]

[a] Laboratório de Química e Função de Proteínas e Peptídeos, Universidade Estadual do Norte Fluminense Darcy Ribeiro (UENF), Rio de Janeiro, Brazil; [b] Núcleo de Desenvolvimento de Insumos Biológicos para a Agricultura (NUDIBA), Universidade Estadual do Norte Fluminense Darcy Ribeiro (UENF), Rio de Janeiro, Brazil. [c] Departamento de Bioquímica, Universidade Federal do Paraná (UFPR), Paraná, Brazil; [d] National Center for Biotechnology Information, National Library of Medicine, National Institutes of Health, Bethesda, Maryland, United States of America.

[#] Corresponding authors:
Fabio L. Olivares: fabioliv@uenf.br
Thiago M. Venancio: thiago.venancio@gmail.com

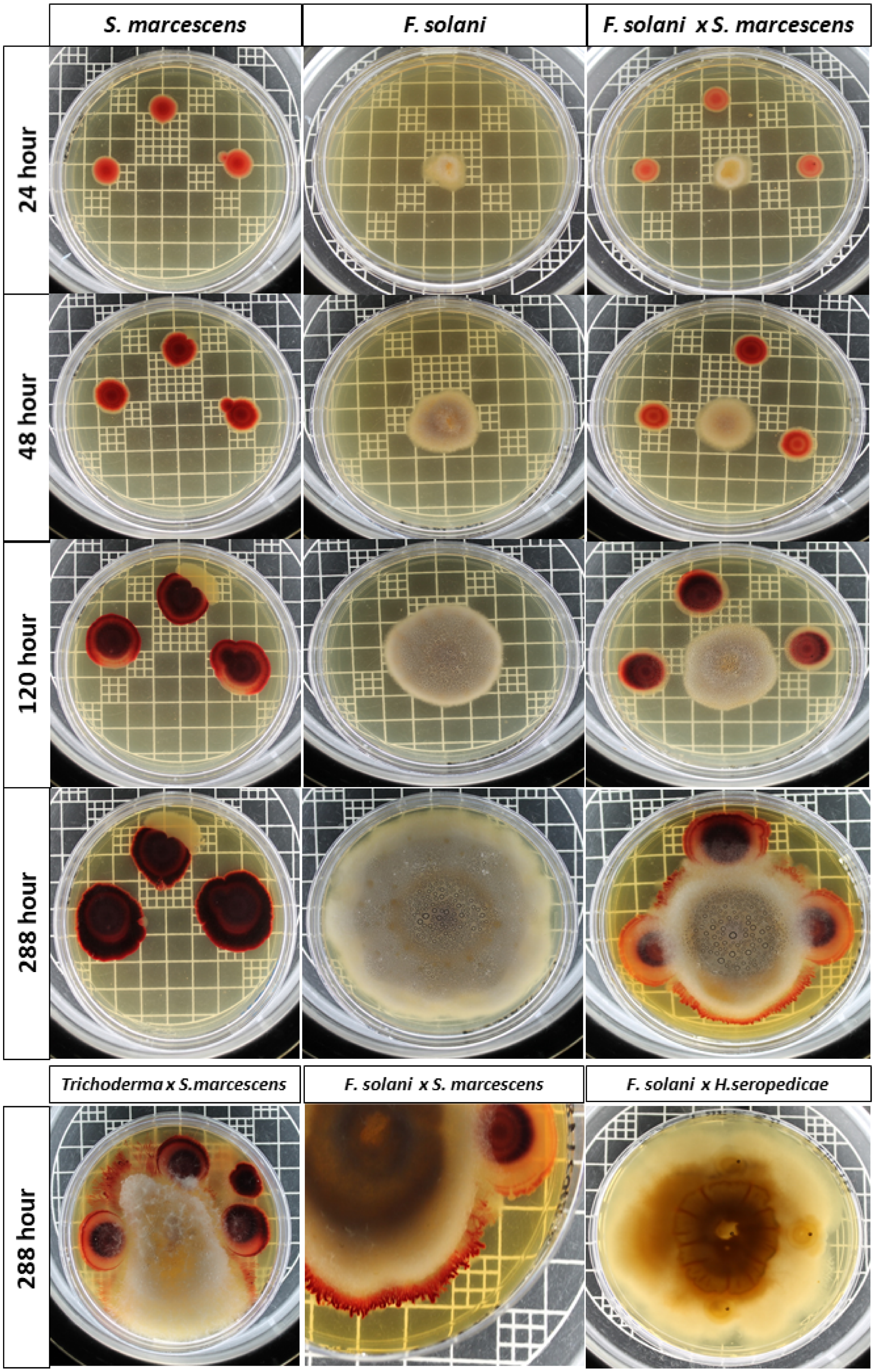

Figure S1: Time-course (24, 48, 120 and 288 h) dual growth of *S. marcescens* UENF-22GI with the phytopathogenic *Fusarium solani* on potato dextrose agar (PDA) solid medium. Note that *F. solani* colony growth (i.e. spread) was reduced by *S. marcescens* UENF-22GI, which in contrast was not significantly affected by the fungus, despite some alterations in the pigmentation patterns. At the bottom line, we show that *S. marcescens* UENF-22GI does not counter the growth of the beneficial saprophytic fungus *Trichoderma* sp.. We also observed a depigmentation of the *S. marcescens* UENF-22GI colony and its spread on the plate surrounding *Trichoderma* sp.. Finally, we used another bacteria species, *Herbaspirillum seropedicae*, to demonstrate that the *S. marcescens* UENF-22GI effects on *Fusarium* are not spurious or merely due to physical occupation of the Petri dish.

a)

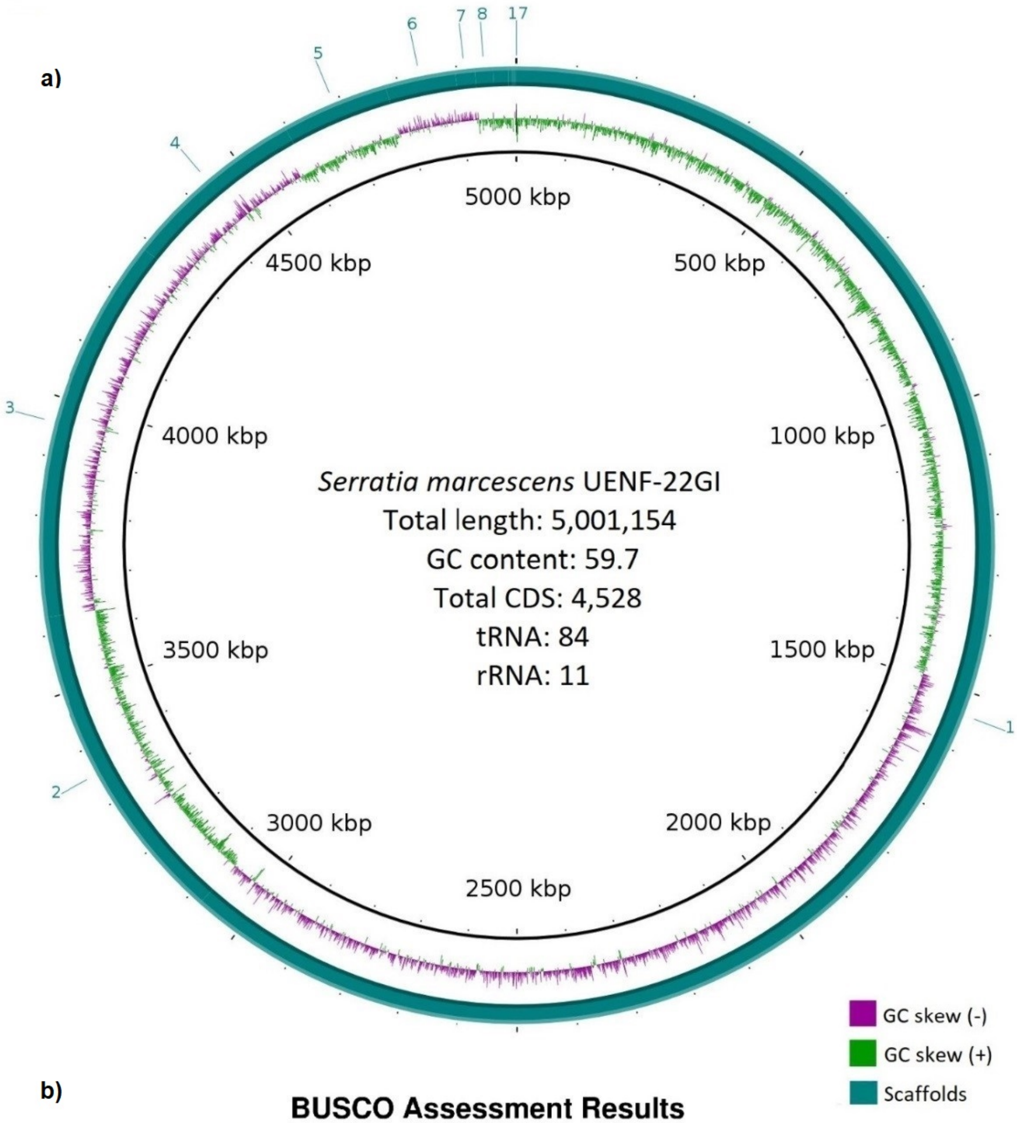

b)

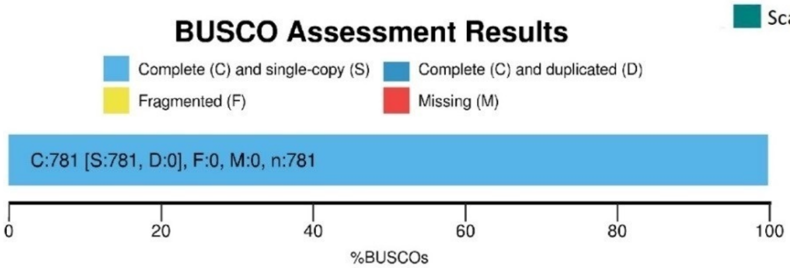

Figure S2: General genomic features of *S. marcescens* UENF-22GI. **a)** Total length, number of protein-coding, tRNA and rRNA genes are represented, as well as the GC skew across the genome; **b)** BUSCO genome completeness assessment using 781 single-copy genes from the *Enterobacteriales* reference dataset.

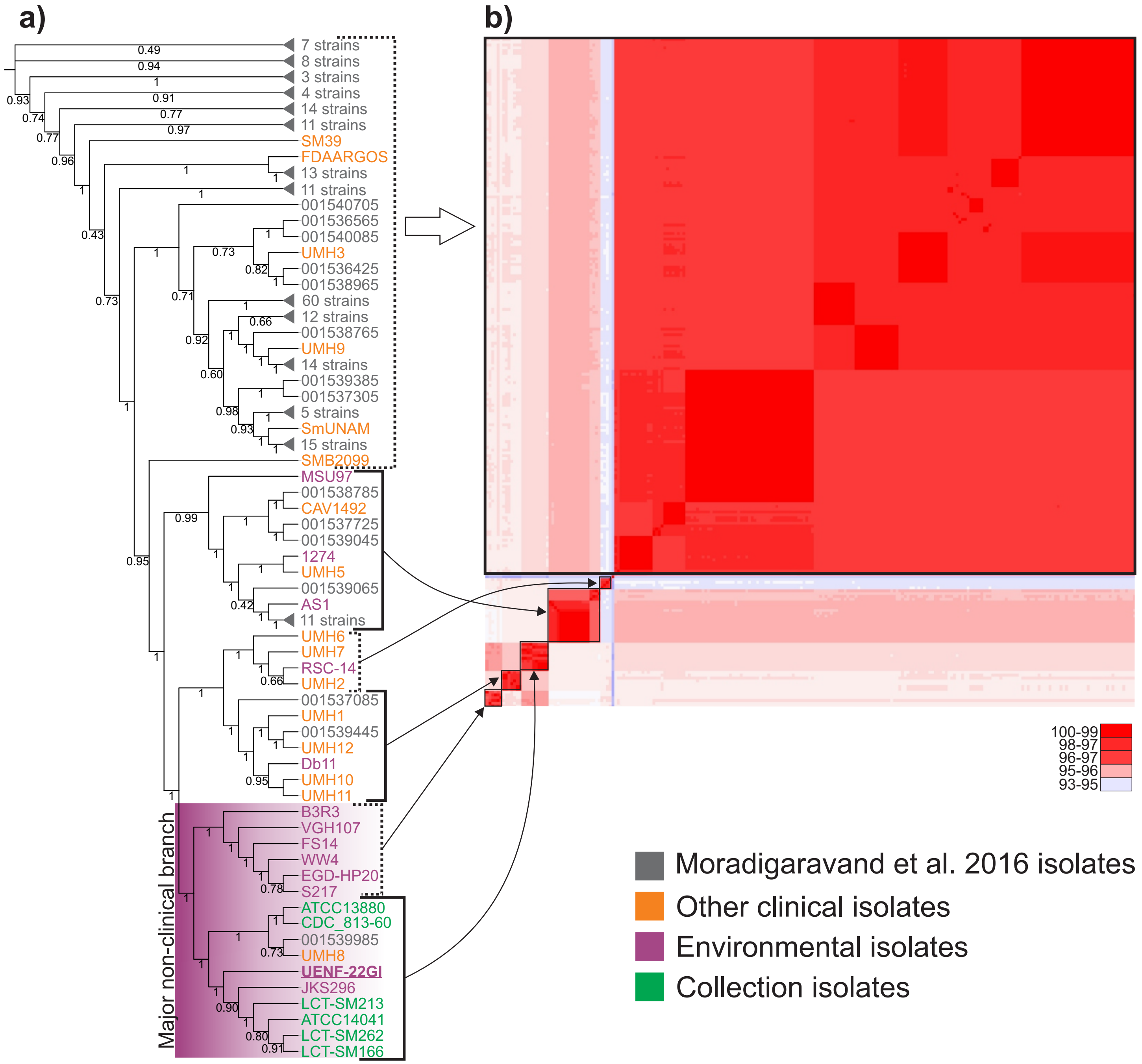

Figure S3: a) Maximum likelihood phylogenetic tree reconstructed with the alignments of the protein products of the 1,815 core genes identified using 238 *S. marcescens* isolates. The tree was built with FastTree 2.1 (https://doi.org/10.1371/journal.pone.0009490). Branch labels represent SH local support values. The purple shaded box delimits the clade containing *S. marcescens* UENF-22GI and is mostly comprised of non-clinical strains; **b)** Clustering analysis of *S. marcescens* strains using Average nucleotide identity (ANI). This analysis also supports that *S. marcescens* UENF-22GI belongs to a mostly non-clinical clade.

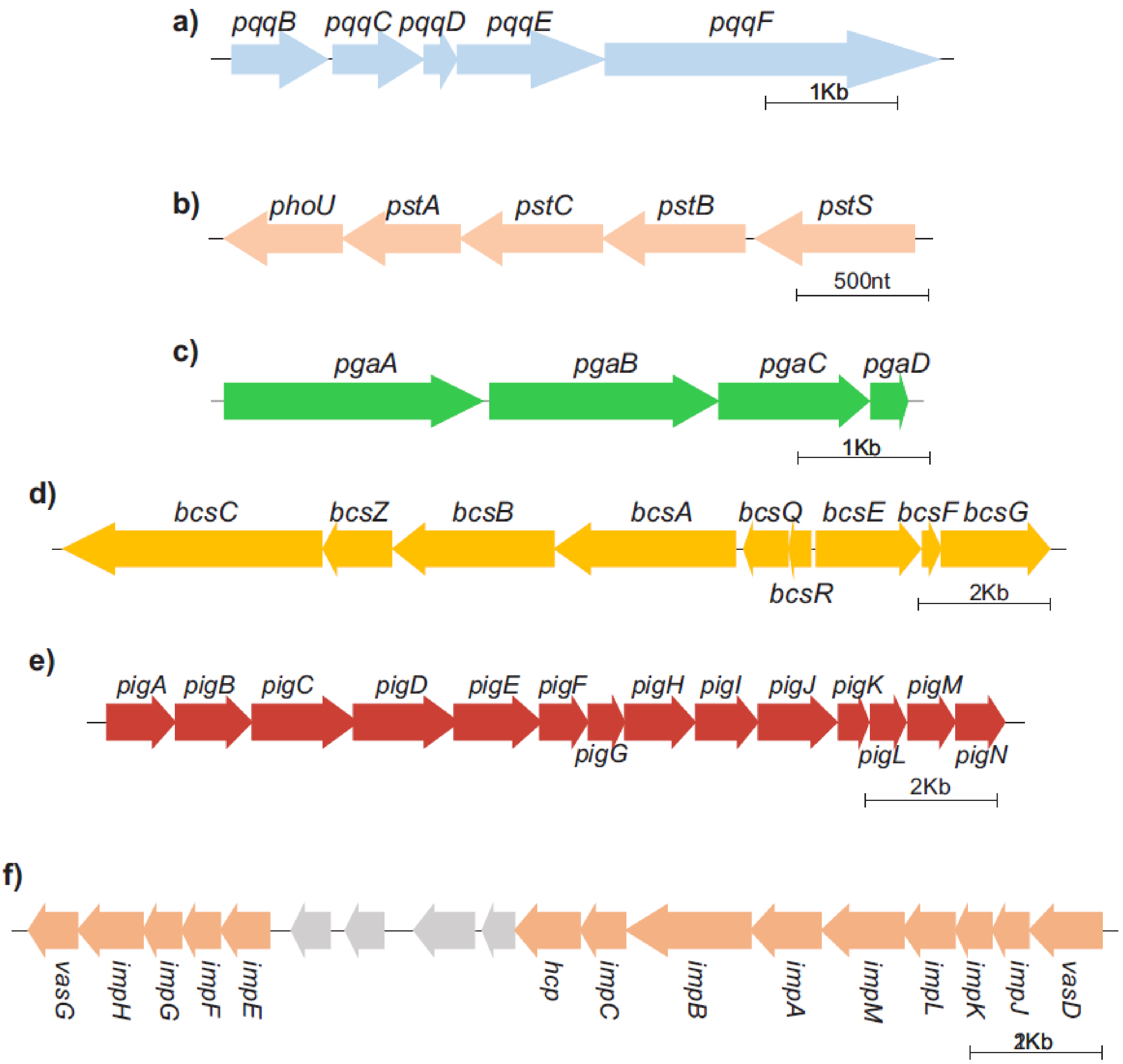

Figure S4: Plant growth-promoting operons found in the *S. marcescens* UENF-22GI genome. **a)** biosynthesis of pqq; **b)** phosphate transport system; **c)** poly-beta-1,6-N-acetyl-glucosamine biosynthesis; **d)** bacterial cellulose biosynthesis; **e)** prodigiosin biosynthesis; **f)** type VI secretion system (genes of unknown functions are in gray).
